# Mindful sharenting: how millennial parents balance between sharing and protecting

**DOI:** 10.3389/fpsyg.2023.1171611

**Published:** 2023-07-25

**Authors:** Michel Walrave, Sofie Robbé, Luna Staes, Lara Hallam

**Affiliations:** ^1^Research Group MIOS, Department of Communication Studies, University of Antwerp, Antwerp, Belgium; ^2^Research Group M^2^P, Department of Political Sciences, University of Antwerp, Antwerp, Belgium; ^3^Department of Business and Law, AP University of Applied Arts and Sciences, Antwerp, Belgium

**Keywords:** sharenting, mindful sharenting, children, parents, privacy, communication privacy management theory, social media

## Abstract

**Background:**

Sharenting, parents’ sharing of personal information about children on social media is becoming increasingly controversial. Its potential risks have drawn some parents to engage in mindful sharenting: parents’ application of strategies to reduce the potentially negative effects of sharenting, as they are aware of the impact sharenting can have on the child’s privacy.

**Objective:**

This study aims to investigate parents’ motives for engaging in mindful sharenting, the strategies they implement and how relatives and acquaintances react.

**Methods:**

In-depth interviews were conducted with eight mother–father dyads in Belgium. At least one of both respondents had to be born between 1980 and 2000 (i.e., millennial parents), having a child aged between 0 and 6 years. Conversations were transcribed ad verbatim, coded in Nvivo, and were analyzed thematically.

**Results:**

The reasons leading parents to engage in mindful sharenting were previous negative experiences they encountered or heard of from acquaintances. In addition, parents aimed to safeguard their child’s privacy and prevent any misuse of their identity or any other forms of aggression. Furthermore, certain parents wish to grant their children the freedom to choose which media content about them is shared online at a later stage in life. As parents are aware of potential benefits of sharenting, they employ strategies to ensure their child’s privacy, while still enjoying the benefits sharenting offers them. These strategies include photographing the child from a distance, the child looking away from the camera, focusing only on a body part, covering the face with an emoticon, blurring the face, or cutting recognizable parts from the photo. However, parents engaging in mindful sharenting are also confronted with questions and negative comments from family members and acquaintances. This makes them feel like they must justify their decision. Moreover, they are sometimes confronted with family members posting identifiable pictures of their child, which leads to privacy turbulence, and parents having to clarify and renegotiate the privacy boundaries concerning image sharing.

**Conclusion:**

Parents deciding to engage in mindful sharenting engage in several strategies to balance between the opportunities sharenting can offer them, the social pressure they experience to post child-related updates, and their objective to protect their child’s privacy. However, some parents face criticism, making them feel pressured to justify their decision and having to clearly explain to family members not to make identifiable pictures of their child available online.

## 1. Introduction

Parenting is a life-changing experience that impacts parents’ individual and social life. The joy, questioning, and issues young parents experience are increasingly also shared online. When parents engage in divulging personal information, e.g., photos, videos, status updates concerning their child, they engage in “sharenting”, a merge between “sharing” and “parenting” (Brosch, 2018). In general, 82% (U.S.) parents declare to have posted information about their children on social media (Auxier et al., 2020). Other research focused on children’s involvement in parents’ decision to engage in sharenting. Research has found that 20% of European children’s (aged 12–16) parents or carers shared personal information about them on social media without asking (Smahel et al., 2020). However, some studies also found parents reflecting on the consequences of disclosing personal information about their child (Cino and Wartella, 2021; Walrave et al., 2022). Some parents take a critical stance towards sharenting and adapt their behavior by not sharing personal identifiable information of their child or by adopting privacy-protecting strategies when engaging in sharenting (Ammari et al., 2015; Autenrieth, 2018). However, until now, research mainly concentrated on parents’ sharenting motives (Latipah et al., 2020), and on adolescents’ perception of their parents’ motives and behavior (Lipu and Siibak, 2019; Ouvrein and Verswijvel, 2019; Verswijvel et al., 2019; Walrave et al., 2022). Scarce research focused on parents’ critical attitude towards sharenting and its influence on their sharenting behavior as a consequence (Davidson-Wall, 2018). The present study therefore strives to tackle this gap in the literature by investigating why, as well as how, parents decide to engage in sharenting while at the same time minimizing sharenting-related risks by exerting privacy-protective strategies. Moreover, as sharenting within an online networked environment makes children’s personal information available to online contacts, the recipients can also share this information with a wider audience. Therefore, we also investigate how parents engage in strategies to limit the audience of the sharented content and negotiate the limits of further transmission of the child’s personal information with online contacts (e.g., family members) that were granted access. Overall, investigating parents’ privacy-protective strategies is relevant as the practice of sharenting contributes to the formation of the child’s online identity (Steinberg, 2017). Further, examining parents’ boundaries towards sharenting is meaningful as sharenting increasingly has become part of online family communication (Damkjaer, 2018).

### 1.1. Transition to parenthood

Becoming a parent is considered one of the most difficult adjustment periods (Bartholomew et al., 2012). Although it brings pleasure and affection, parents feel they must perform and meet societal expectations (Collett, 2005). Moreover, caring for the child leaves parents with less time for social contact and leisure activities. As a result, new parents often experience social isolation (Brosch, 2016). Sharenting can contribute to parents’ social capital, resources that are built through relationships with others. Social capital is crucial for new parents to adjust to parenthood (Bartholomew et al., 2012). Two forms of social capital can be discerned: bonding and bridging social capital. Bonding social capital exists within networks with strong ties, and involves a high degree of trust, intimacy, and emotional support (Davidson-Wall, 2018). Especially family and close friends provide this form of connective social capital. Bridging social capital occurs within networks of weak ties and is primarily based on gaining new perspectives and information (Putnam, 2000). For example, acquaintances made through social network sites, colleagues, and other individuals met in professional, or leisure activities are responsible for bridging social capital. These ties also include online contacts with other parents who simultaneously or recently went through the transition to parenthood and can thus share useful information with each other. Expectant and new parents find and support each other increasingly on online platforms which can serve as resources for new parents to deal with the high demands of parenting (Bartholomew et al., 2012).

### 1.2. Conflicting self-presentation

In addition to the need for social support, advice, and belonging, parents turn to social media for self-representation (Blum-Ross and Livingstone, 2017). They do this by presenting themselves on social media based on the personal data they share, and how they present this information online (Holiday et al., 2022). However, parents experience a tension between the presentation of the “individual self,” i.e., the parent as an individual, and the “relational self,” i.e., the role as a parent exercised with and for their children and family, and in the face of the (online) community they are integrated into (Blum-Ross and Livingstone, 2017). Parents’ individual online self-representation is a reflexive practice where every decision regarding one’s identity can be seen and can be controlled. These decisions concerning the online image that parents want to portray of themselves can be seen as a form of impression management. Impression management assumes that people have some degree of control over the way in which they are perceived by the public (Leary, 2001). This is usually guided by desires about how one would like to be and how one is expected to be, based on social roles one performs in society (Ouvrein and Verswijvel, 2019). Parenting is one of the social roles which guides one’s self-representation (Collett, 2005). By sharing information online about their children, family activities, and how they cope with the challenges of parenthood, parents seek to demonstrate their parenting competencies (Walrave et al., 2022). This is referred to as indirect self-representation, where individuals use others with whom they are closely related, such as in this case their children, to shape their own representation (Ouvrein and Verswijvel, 2019). Parents further engage in sharenting as they want to share important and mundane moments of their child’s life. Parents often illustrate how their child grows and changes physically as well as how it performs in school or spare time related activities (Brosch, 2016). They also emphasize milestones of their child’s life, such as birthdays, graduation, family holidays and other common activities (Kumar and Schoenebeck, 2015). Their child’s digital visual narrative is created and shared with family members and acquaintances. By doing so, parents chronicle their child’s development for current online contacts to view and comment on, but also to cherish these memories for the future. Parents also want to show their pride and highlight important events and accomplishments for their online contacts to witness. They also post these achievements and family moments online to show the role they play as a parent, and to feel supported and validated through the comments and likes they receive (Kumar and Schoenebeck, 2015; Blum-Ross and Livingstone, 2017; Davidson-Wall, 2018; Walrave et al., 2022).

However, by posting personal information about their children, parents also shape the digital presence and identity of their children before they themselves are active on social media (Latipah et al., 2020). This parental impression management of the child is potentially at odds with the child’s developmental task to build an autonomous identity (Davidson-Wall, 2018). However, the online identity which parents form for their children through sharenting, may not align with how adolescents want to represent themselves online. Therefore, young people may engage in privacy management strategies, protecting personal information so parents or others may not access and further transmit specific information (Verswijvel et al., 2019; Walrave et al., 2022).

Privacy management strategies and impression management are, however, in a dialectical relationship because the information that is suitable for self-representation may be information that can harm the individual’s privacy the most. The trade-off between the two becomes increasingly difficult when individuals share personal information with multiple, asynchronous audiences in online environments, where the boundary between public and private is increasingly blurred (Picone, 2015). According to research, the merging and collapsing of social spheres on social media can cause some conflict in the account of the holder’s perception (Binder et al., 2009; Marwick and Boyd, 2011). This tension results from the challenge of concurrently deciding which personal information is suitable to transmit across various social realms. To mitigate risks of personal data disclosure on platforms where different social circles are co-present, users can employ several strategies, such as: the use of privacy settings to differentiate the level of access to one’s data between social media contacts, choosing particular communication channels to disclose specific information to specific contacts (e.g., instant messaging apps instead of social network sites), avoiding conflicts of context by not disclosing information and thereby employing self-censorship, or discussing clearly with others not to further transmit the entrusted information (Lampinen et al., 2009; Walrave et al., 2012; Heirman et al., 2016).

### 1.3. Millennial parents

The potential conflicts between parents’ sharenting and adolescents’ self-presentation, may be influenced by parents’ own online experiences. Parents could have grown up in a period before the development and rise of social media or, by contrast, could have been raised in the public’s eye, as their parents engaged in sharenting. More particularly, sharenting may have a different context for millennial parents. These parents were born between 1980 and 2000, have grown up with social media (Latipah et al., 2020), and are therefore referred to as the digital natives (Autenrieth, 2018). As they become parents themselves and raise their children in a digital media culture, they can be stimulated to record and share activities digitally (Putri et al., 2021). Moreover, the creation of photographs and videos has improved tremendously over the years. In the analog era, the number of photographs was limited by the cost of production and the effort involved. Today, most phones include a high-quality camera, allowing parents to capture images of their children anytime, anywhere. This fleeting use is further strengthened by the long-lasting conservations and easy sharing of these images on social media (Prensky, 2001; Autenrieth, 2018). Moreover, today, sharenting is seen as the social norm in this digital age (Blum-Ross and Livingstone, 2017; Siibak and Traks, 2019). New parents are often encouraged to share images and stories of their own experiences as parents and details about their growing-up children (Blum-Ross and Livingstone, 2017). Therefore, managing and controlling the online flow of information related to both parenting and family life constitute a responsibility for parents (Kumar and Schoenebeck, 2015). Parents need to balance between privacy-protective behaviors, as they are the guardians of their children’s personal information, the benefits sharenting has to offer them, and how to meet societal expectations (Wagner and Gasche, 2018). Millennial parents’ motives to engage in sharenting center around getting affirmation and social support through likes and comments (Robiatul Adawiah and Rachmawati, 2021). This feedback confirms young parents in their competence in taking care of their children. As engaging in sharenting is normative among today’s parents, young mothers have been found to indicate their online contacts make snap judgments when they deliberately choose not to engage in sharenting (Siibak and Traks, 2019). Some parents, therefore, indicate that they feel pressured by family and friends to share children’s photos online (Ranzini et al., 2020).

### 1.4. Reactions against sharenting

At the same time, concerns have increasingly been voiced about sharenting’s potential drawbacks. These can include both privacy and security risks. For instance, digital kidnapping is a phenomenon where a stranger steals photos of a minor from the Internet and posts these photos as if they were from his own child. Other research has observed how children’s photos are plucked from social media platforms and then shared on child abuse image websites (Otero, 2017; Garmendia et al., 2021; Williams-Ceci et al., 2021). Furthermore, through sharenting, parents are forming their children’s online identities without their consent (Steinberg, 2017). This can have a negative impact on the development of children’s personalities (Verswijvel et al., 2019). Because children are widely portrayed by their parents, they do not have the opportunity to create their own online identity. For some children, the content that parents think is appropriate to share on social media may be sensitive, or some content may lead to negative reactions. As a result, children may face cyberbullying (Robiatul Adawiah and Rachmawati, 2021). Besides, images may stay online or resurface later in the context of (potential) employers’ cybervetting (Walrave et al., 2022). In sum, the choices parents make today may have long-term consequences for their children (Leaver, 2020).

Moreover, through sharenting, children may grow up holding a very different concept of privacy (Davidson-Wall, 2018). It may seem normal for some of them that their personal information is made public online. On the other hand, certain children who are growing up may become increasingly sensitive to their own privacy and the privacy of others when faced with their parents’ sharenting. This sensitivity may arise from their firsthand experience of personal information being shared without their involvement in the decision-making process. As potential consequences of sharenting are increasingly discussed and sometimes become apparent for parents and children, sharenting is becoming subject to debate (Autenrieth, 2018). In some countries, awareness raising campaigns are informing parents about the potential drawbacks of sharenting, stimulating them to engage in privacy-protective strategies and, when possible, discuss the sharenting decisions with their child (Diebel, 2022).

### 1.5. From sharenting to mindful sharenting

Some parents are deliberately choosing not to share information about their child or, when they do, employ strategies to limit the risks of sharenting. Autenrieth (2018) describes this as “anti-sharenting”, specific practices to make their child unidentifiable when sharing their pictures online. In the tension between the need to put pictures of their children online while leaving as few visual traces as possible, parents have developed new photo practices. For instance, parents focus on the photographic and spatial context of the image, rather than on the child. These practices allow them to show their children on social media while maintaining some form of anonymity (Davidson-Wall, 2018). In photography, this is also known as the “anti-selfie” (Tifentale and Manovich, 2018). In this type of photograph, the person in the selfie becomes part of a situation rather than being depicted as isolated.

However, we state that the concept anti-sharenting could signal something different than employing strategies to reduce the risks of sharenting, as the prefix “anti” is used to indicate someone is against someone or something (Cambridge Dictionary, 2023). Parents who choose to employ strategies to protect their child’s privacy on social media, are still engaging in sharenting and, therefore, not necessarily opposed to it. By adapting the pictures, some parents ensure that their child(ren)'s privacy is not compromised while, at the same time, they can enjoy the benefits of sharenting (Wagner and Gasche, 2018). These parents cannot be seen as opponents of sharenting. Therefore, we propose to coin this behavior as “mindful sharenting”, which consists of the application of strategies by parents to reduce the potentially negative effects of sharenting, as these parents are aware of the impact sharenting can have on the child’s privacy. Moreover, this well thought-out form of sharenting is further inspired by the characteristics of “mindful parenting” a way of parenting that adheres to the principles of mindfulness (Geurtzen et al., 2015). Mindful parenting is characterized by five key aspects: (1) listening with full attention (paying quality attention and being able to accurately perceive what the child is trying to communicate), (2) nonjudgmental acceptance of the self and the child (appreciating the characteristics of the child, acknowledging that there will be challenges, mistakes, and unmet expectations, but also setting clear standards for the child’s behavior), (3) emotional awareness of oneself and the child (less dismissing and more responding to the emotional need of the child; correctly recognizing own and the child’s emotions), (4) parenting in accordance with one’s goals and values, and (5) compassion for oneself and one’s child (positive affection in the parent–child relationship) (Shorey and Ng, 2021). In relation to mindful parenting, engaging in mindful sharenting can be inspired by specific goals and values that lead to parents’ sharenting decisions. In doing so, parents are aware of their motives and purposes for engaging in sharenting as well as the impact sharenting can have on the child and themselves. They consider the potential consequences for their child and, depending on their child’s age and agency, the child’s own emotions and opinions before engaging in sharenting. In other words, the act of sharing or not information about their child is fuelled by parents’ consciousness of their own objectives for engaging in sharenting and its potential impact on their child, now or in later life. Mindful sharenting therefore also includes privacy-mitigating strategies to lower the (short- and/or longer-term) risks of sharenting for their child.

The specific strategies parents can implement reduce the focus on the child and emphasize the photographic and spatial contexts of the images. There are five types of photographic practices that focus on making the child unidentifiable. First is the “disguised child” (Davidson-Wall, 2018), in which attributes such as a scarf or glasses are used to make the child unidentifiable (without specific photo processing). A second way is to photograph the child from a distance to make it less identifiable (“the faraway child”) or photographed from behind and in a wider context (“the child from behind”). What also can be done is to photograph a specific body part of the child, such as a hand, or foot (“the parted child”). This will keep the child unrecognizable to unknown viewers. In addition to photographing only a particular body part, one can also choose to photograph the child when it is looking away from the camera. Finally, one can digitally edit the photo by using, among other things, an emoticon to cover the face (“the digitally processed child”) (Davidson-Wall, 2018). Beyond the method of taking the photo and editing it, it is also important for some parents to avoid other potential identifying information. For example, some use only the initials of a child or a pseudonym. Parents can also adjust the reach of their social media posts. By using private groups on Facebook or Instagram, stories are shared with a select group of followers. Parents can also decide to share pictures through messaging apps such as WhatsApp or Messenger. Taken together, these strategies are employed by parents to mitigate the disadvantages of sharenting (Ranzini et al., 2020), while enjoying the benefits sharenting offers them (Autenrieth, 2018).

### 1.6. Communication privacy management theory

Parents’ efforts to reflect on sharenting’s consequences and, when possible, to include the child in the decision-making process concerning the sharing of information about the child, can be related to the Communication Privacy Management (CPM) theory (Petronio, 2002). In general, CPM theory helps to explain individuals’ management of disclosing and protecting personal information (Petronio and Child, 2020). In the case of sharenting, it can shed light onto how parents, children and family members, as social media users, evaluate their privacy needs and develop rules for social media use (Child and Petronio, 2015) and, in the present context, for engaging in sharenting (related) practices. A key tenet of CPM theory is that individuals believe they own personal information related to them, and have the right to control its dissemination. Ownership is symbolized by metaphorical privacy boundaries, wherein personal information is kept, or where others are brought into the privacy boundary, when they become (authorized) co-owners of the personal information (Child and Petronio, 2015). Applied to sharenting, parents decide which information of their (underage) child is disclosed online and with whom. Recipients become co-owners who, at their turn, control the information’s further dissemination. In line with the theory, parents tend to establish rules about privacy boundaries, which the extended circle of family and friends must adhere to, in order to maintain ownership of personal information about the child (Cino and Dalledonne Vandini, 2020).

Parents’ privacy rules may be formed through observation (e.g., of other parents’ (over)sharenting) or their experience as a child, more particularly, their parents’ sharenting practices and how it forged their vision and practices concerning sharenting. The establishment of these privacy rules, and their alignment among individuals, are an ongoing communication process in which there is a trade-off between the risks and benefits of disclosing information about the child (Walrave et al., 2022). Parents can use privacy rules to guide other co-owners of the child’s personal information, to manage the access to this information. In sum, privacy boundaries are singular (around personal information of one person) as well as collective (when personal information is co-owned by others (e.g., family members)) (Petronio and Child, 2020). Privacy boundaries are managed through three types of privacy rules. First, privacy boundary linkage rules focus on decisions co-owners make concerning who else can know the personal information (applied to sharenting, parents stating their child’s picture may not be transmitted to a specific family member). Privacy boundary permeability rules determine which type of information and how much information can be disseminated outside the set privacy boundary (e.g., adolescents asking their parents not to share pictures of them when they were a child or other pictures that they could find embarrassing). Finally, privacy boundary ownership or control rules determine the rights of co-owners to make their own decisions concerning the further dissemination of information (e.g., parents sharing photos in a private WhatsApp group, and asking its members to first ask them if they want to further transmit pictures to others) (Petronio, 2002; Child and Petronio, 2015; Petronio and Child, 2020). In other words, parents engaging in mindful sharenting may want to control co-owners’ further dissemination of the entrusted child-related information. Therefore, they might more explicitly discuss with others the degree, or conditions, of privacy boundary permeability.

Yet, when privacy rules are not made clear, or openly discussed within the couple who raises the child, their family and circle of friends, there is a chance that privacy boundaries will be crossed. This can lead to privacy turbulence, which occurs when there is a violation, intentional or not, of the privacy rules, control or ownership that were established (Petronio, 2002). These privacy violations can affect the core of the relationship between friends or family members (Steuber and McLaren, 2015). In addition to the negative consequences and feelings related to these privacy breaches, it also provides an opportunity to (re)explain or recalibrate the privacy rules, in order to prevent future privacy turbulence (Petronio and Child, 2020).

As demonstrated above, the process of engaging in sharenting can be situated and explained through CPM theory. Previous research has focused on negotiations between parents and adolescent children (Walrave et al., 2022). However, little is known about the decision-making process of young parents who decide to employ strategies to mitigate the disadvantages or risks of sharenting (Wagner and Gasche, 2018). Therefore, the present study focusses on the following research question: What are the motives of millennial parents (born between 1980 and 2000) with young children (0–6 years) to engage in mindful sharenting? (RQ1).

New parents are encouraged to share photos of their children online. However, the choices parents make, may have long-term consequences for their child(ren) (Leaver, 2015). Parents must thus, in this digital age, balance between privacy-protective behaviors to protect their child(ren) and enjoying the benefits sharenting offers them. The decision of young parents to engage in mindful sharenting may be fuelled by observing sharenting behavior of others or by their own experiences that led parents to discuss and reconsider their sharenting behavior. Therefore, the second research question of this study is: Which situations or observations have led to parents’ decision to engage in mindful sharenting? (RQ2).

When they engage in sharenting, parents can employ different strategies to protect their child’s online privacy (Autenrieth, 2018). In addition to applying strategies when photos are shot, parents can also choose to avoid sharing identifiable information. They also can limit the reach of these pictures on social media, by using private groups or limiting the reach of their posts through more stricter privacy settings. Moreover, parents can establish explicit and implicit rules to discern what can and cannot be posted online about their child (Ammari et al., 2015). This is not only important for the parents themselves, but also for the extended family so that they can understand and handle these rules (Cino and Dalledonne Vandini, 2020). To further investigate these strategies, this study also focuses on the following research question: What strategies do young parents who engage in mindful sharenting employ to protect their child’s online privacy and how do online acquaintances react to it? (RQ3). More particularly, we investigate how parents negotiate with each other and family members the sharing of personal information concerning their child, considering how they engage in their child’s privacy protection. In situations where parents’ and family members’ vision and practices conflict, how do they react when privacy turbulence occurs, and further discuss, or possibly, recalibrate privacy boundaries? (RQ4).

## 2. Methods

### 2.1. Participants

Eight semi-structured interviews with mother/father dyads (16 participants in total, mean age: 31) were conducted in Flanders, the Northern part of Belgium, between March and April 2022 (for an overview of the participants, see Table 1). Both the mother and father of the child(ren) were interviewed together to get insight in the mindful sharenting decisions they take as a couple. Participants were recruited through specific parental Facebook groups and acquaintances based on four criteria. At least one of both parents needed to be Flemish, had to be born between 1980 and 2000 (i.e., millennial parents), were parent of a child aged between zero and six and said to engage in mindful sharenting which the authors defined as limited and consciously sharing of information about their child(ren) on social media. Since the corona pandemic was not over yet, seven out of eight interviews took place online via Microsoft Teams. Interviews lasted on average 46 min. During the last interviews no new topics emerged from the conversations signalling that data saturation was reached, the point in the data collection when no supplementary insights or topics were identified and indicating that continuing to collect data would be redundant. The collected data captured the diversity, depth and nuances of the issues studied (Hennink and Kaiser, 2022).

**Table 1:** Background information of interview participants.

	Pseudonyms	Age	Profession	N of children	Age of children
Couple 1	Anne and Tim	28 and 28	Preschool teacher and forman	1	8 months
Couple 2	Karen and Johan	29 and 30	Speech therapist and insurer	1 (+pregnant)	2 years
Couple 3	Evelien and Frank	30 and 34	Branch manager and warehouseman	1 (+pregnant)	2 years
Couple 4	Lisa and Bruno	29 and 30	PhD Candidate and port worker	1	6 months
Couple 5	Tina and Mats	35 and 33	Actors	1	3 years
Couple 6	Sara and Daan	35 and 37	Officials	2	6 and 4 years
Couple 7	Elise and Joris	29 and 29	Nurse and educator	2	2 years and 5 months
Couple 8	Sanne and Tijs	30 and 30	Occupational therapist and service technician	2	2 years and 2 weeks

### 2.2. Interview procedure

Interviews were audio recorded and proceeded as follows. First, the interviewer gave a brief introduction of the research topic. Respondents confirmed their voluntary participation and gave their permission to make an audio recording. The interviewer also reminded the couples that they had the right to stop the interview at any time. The participating couples signed a consent form they received along with the information form that explained the objectives of the study and the respondents’ rights (in terms of data protection and the right to withdraw from the study). This procedure followed the guidelines of the University of Antwerp. The interview consisted of four parts. After the introduction, the interviewer asked the couples about their parenthood experiences and how they perceive their role as a parent (part 1). Next, participants were probed about their social media usage (part 2). These introductory questions focused on their motives for being active on social media, the changing role of social media before and after pregnancy, and their opinion about sharenting. Next, the interviewer moved to the core of the interview, the couples’ mindful sharenting behavior by asking, e.g., why they engage in mindful sharenting and how friends or family members cope with their decision to cautiously share information of their child online (part 3). At the end of the interview, the interviewer asked to show some media content they shared about their child(ren) to illustrate their mindful sharenting behavior (part 4). Finally, the couples were thanked for participating and reminded that they could contact the interviewer for further information at any time.

### 2.3. Data analysis

After conducting the interviews, conversations were transcribed ad verbatim and coded as soon as possible in Nvivo12. The coding of the interviews followed an inductive approach and were analyzed thematically in line with the six-phase process (Braun and Clarke, 2012). Before coding, first transcripts were read carefully to verify that data saturation had been reached. Next, one researcher identified every answer that was given by the participants and provided them with a code. Codes referred to different parts of the interview varying from the motives for sharenting to tactics to engage in mindful sharenting. Each answer was coded on sentence level so that more than one code per answer could be used. After coding, all authors read the eight interviews and preliminary findings were discussed. To increase the validity of our study, quotes were selected by the authors to illustrate our findings. All quotes were translated from Dutch into English and were kept as close as possible to the original expression of the participants. To guarantee the anonymity of the participants, this study makes use of pseudonyms. The names of children were changed by the initials of the child.

## 3. Results

### 3.1. Context of the decision

The eight couples interviewed all consciously chose to share photos on social media of their children where they are not identifiable. What came back from the interviews was that parents themselves, or someone from their circle of acquaintances, had already experienced negative consequences of sharenting. For example, one couple experienced that a picture of their niece was stolen by a colleague of the grandmother. This person used the photo of the niece on his Christmas card. Another mother was confronted with a fake account of a man in a Facebook group for young mothers. In this group, photos and videos of young children were exchanged.

“In a mom group that I’m in with thousand members, they discovered last year that there was a fake account of a man that was just there to see the pictures. So that’s thousands of moms sharing pictures of their babies, which that guy had access to. These include pictures of children in their diapers, and in their swimming trunks. This makes me think that you cannot protect your child that way” (Mother, couple 8).

A father participating in the study told that, when he was younger and involved in a youth movement, pictures were stolen from the movement’s website and found on a child abuse website.

“When I was still in the chiro [Belgian youth organisation], there was someone on our child movement leadership team who pretty much kept the pictures of the kids on a photo website that were shared publicly so that all the parents could access them. Then there was a storm of scandals, as there were photos found on a child porn website in Thailand” (Father, couple 7).

These experiences deliberately made some parents decide to only share photos of their children where they are unrecognizable, or they try to share them selectively (through closed accounts). In addition to the negative experiences, one couple also said they do not share much about themselves online. Therefore, they also rarely share photos of their child. When they do so, they use pictures where their child is unrecognizable. All interviewed couples declared they made this decision before the child was born. Often, one of the parents came up with the idea, and the other partner understood the reasoning behind it and was convinced. The mothers of couple 1 and couple 7 thought it was important that they could still post something occasionally but unrecognizable.

“Yes, for me, that is okay - although we are a couple that is very communicative. If something is put on the table and the other does not agree, we immediately try to find a common ground. We may occasionally share pictures, but then protected” (Mother, couple 1).

Couple 2 and couple 6 initially chose not to share photos. Couple 2 went one step further. They would not even forward photos so that no one could further distribute photos of their child, especially because of negative stories they heard of. Then they loosened these rules.

“Things that happened, stories that we heard from, pictures that really end up in these crazy places. Yeah, that’s why we chose that. In the beginning, then made very hard - very strict lines and, afterward, a little less…” (Mother, couple 2).

Some couples who initially decided not to share their child’s picture online, changed their decision during the COVID-19 pandemic. In March 2020, the first complete lockdown made physical meetings impossible. As a result, it was difficult for family members and acquaintances to see children of friends and family growing up. Therefore, some parents changed their decision not to post photos on social media to sharing pictures where their child is unrecognizable and forwarding photos in restricted circles.

“Because especially the oldest was born in that lockdown, we do have one private Instagram account that only people can be on, that we allow” (Mother, couple 8).

Some parents try to discuss at an early stage with their family members and friends that they want to oversee which pictures of their child would be shared with whom. As the mother in couple 2 states:

“In the hospital they [family members] were all taking pictures. Then we clearly stated, “this is only for your personal use, OK? Keep it on your mobile phone and do not send it to the rest of the world”” (Mother, couple 2).

All parents chose to engage in mindful sharenting. They find it important to be able to post something from time to time about their children, because this is an important part of their lives. The parents also indicated that they experience social pressure to share pictures of their children on social media. Some parents feel the need to engage in sharenting for affirmation, support, and sharing joyful moments. So, these parents also feel a need to post photos in order to experience these benefits. Nevertheless, these parents are trying to do so in a way their children do not experience any disadvantages.

“I’ve experienced that a few times. I was so super proud, when our kids do something or so, and thought “oh, too bad we made that decision.” I wish I could show it now in all its glory, like “Look, he can do that already!” I still do not regret our decision. You have to think more about “I think this is a very beautiful moment, I want to take a picture of that and how do I take a picture without him being recognizable?”” (Father, couple 7).

Some parents explicitly highlight the difference between their generation and the generation of their parents, aunts and other (older) family members. As our respondents grew up with social media, they explicitly mention that they share less on social media.

“Our mother, her friends, and the aunts of our child, they put everything online, while our generation puts less information online” (Mother, couple 2).

Parents even testify that their parents have difficulties understanding their decision and that some specific arrangements had to be made to give the grandparents the possibility to share photos in their social circle, without putting pressure on the parents’ decision.

“Our mum did have some trouble with it, but I think also rather so out of pride. She just wanted to share a lot about her grandchild with her friends. We had to say “Yes, just do not do that in public. Share it in a conversation with one person or so, but just not with too many people at once.” We had to explain that occasionally, but then she understood it. She respected that” (Father, couple 7).

### 3.2. Motives of the decision

The main motive that emerged during the interviews was to protect the privacy of the child. After all, a photograph contains a lot of sensitive information. Furthermore, the photographs can also be shared on child abuse websites, or children may become the target of cyberbullying or other forms of aggression. Some parents also refer to rapid digital developments related to face recognition and what it may lead to in the future. Parents also state it is not necessary that, so to speak, the whole world sees their child growing up. The most important people see the child growing up in real life. Moreover, some parents do want to prevent their child associating them with taking pictures of them instead of engaging in other interactions.

“Won’t your little one be more used to seeing your smartphone camera instead of yourself as a parent? That they will have the feeling “Oh, you are just looking at me through that little screen.” They will not find that attractive, as they are always seeing you with your cell phone in your hand instead of just being busy with them” (Mother, couple 4).

The same mother makes a comparison with other activities, people from a concert audience who are filming instead of enjoying, which makes her similarly question why they are not in the moment.

“I have the same reservation at concerts or festivals. People who are filming everything. Then I think “just look at what is there, instead of looking through your cell phone.” So, I think that lacks authenticity. I really wonder why they do it. Not necessarily in a bad way, but I’m curious about what’s behind it, that continuously sharing” (Mother, couple 4).

Some parents make this decision from the start. When they observed some parents sharing ultrasounds of their future child, they found this odd and would not do that. As the following father expressed his doubts about sharing photos of the unborn child.

“It just does not feel natural to me to do such intimate things like - there’s a baby in your belly and it’s growing there. It’s just on the borderline of having or not having life, you know. It is the essence of our existence. Life is growing inside you, you pass on life. Such a picture of a baby in the belly, then sharing it on the Internet. I find that so intimate” (Father, couple 5).

Furthermore, parents are also aware of the fact that, as soon as a picture is online, they no longer have control over the reach of that photo’s audience. Another motive that emerged was that parents want to allow their child to choose what media content they share about themselves on social media. They are convinced it is, in fact, not acceptable to start distributing photos on social media without the child’s consent. The child is not asking for its image to be publicly displayed. In this way, parents want to show how important privacy is and that they are aware of the impact sharenting can have on children.

“We want to give our children the choice, later when they are older, to share images about themselves, if they want to share them” (Mother, couple 6).

Or as another parent points out:

“If he later wants to be on Instagram, what I will not advise, then he’s free to do it. He can put whatever picture he wants online. But I will not stimulate that. I will not, before he can give permission, put him on that. This is for me the most important because he did not ask for it” (Father, couple 5).

Some parents refer explicitly to their responsibility to educate their children concerning the potential risks when they engage in online activities and disclose personal information. Moreover, as one respondent highlights, they as young parents have grown-up with social media.

“Always stay aware of any activity that is performed on the Internet, as long as they are underage, under 18. Yes, really do pay attention to it. Parental supervision, what are they doing, who are they chatting, texting or calling with. And also, when they are of age 18 that they know they can come to us. The advantage is, we have grown up in this era, so we have seen social media and the digital world evolve” (Father, couple 3).

### 3.3. Strategies for mindful sharenting

The parents employ several strategies to protect their children’s identity as much as possible from the disadvantages of sharenting, while at the same time enjoying the benefits sharenting offers them as parents. The strategies they primarily employ focus on the spatial context rather than on the child (cf. Table 2). The child is photographed from a distance. Or the child is photographed from the back. Half of the parents also posted photos of certain body parts of the child, such as a foot, a hand, or an ear. Furthermore, sometimes an emoji is placed over the child’s face. One couple of parents (couple 8) sometimes blurs the face or cuts recognizable parts of the photo.

**Table 2:** Mindful sharenting strategies adopted by parents.

	Picture from a distance	Look away from the objective	Focus on a body part	Emoji on the child’s face	Shield recognizable body parts	Blur the child’s face	Referring to the child without a photo of the child
Couple 1			X	X			
Couple 2	X	X	X	X			
Couple 3		X		X			
Couple 4							X
Couple 5		X	X	X			
Couple 6	X	X					
Couple 7	X	X					
Couple 8	X	X	X		X	X

“When A. is lying with me, I share a picture of his hand and his ear. You can only see that. For instance, I say that it was a “hang day” because he had his shots. Because I want to share this with people who follow me, but I’m never going to fully portray him” (Mother, couple 8).

One of the couples had a very particular way of sharing photos. They share photos in which their child cannot be seen, but the photos are posted with their child in mind and referring to the child. For example, they post pictures of socks, a toy, or an abstract picture of a baby carriage. This maintains the anonymity of the child while including details that are important to express a situation or refer to the child.

“Then I do put text with it, in which it is made clear that it is posted with A. in mind or that it does concern her” (Mother, couple 4).

Three couples indicated that the photos were taken spontaneously, and it was decided afterward which ones they were going to share. A parent, showing a picture to make his point, said the following:

“This photo, for example, is one made where I had to think of other points of view. I would initially think, “I want to pull that so frontal because then you really see he already has his head up really well,” but then I start thinking “yeah, we do not want the face on it, which side can I photograph him?,” so that it is clear what he is doing, but without showing his face” (Father, couple 7).

The most important rule to keep in mind, according to the parents, is that the child should not be recognizable in the photos or videos posted online. Furthermore, parents also apply other privacy protective measures. For example, some parents started a private WhatsApp group, in which photos of the child are shared with family members and close friends. When parents do not do this through a WhatsApp group, they use another application such as Family Album or Google Photos. These are photo albums shared online, where parents have control over who has access to their child’s pictures.

“Family Album is our alternative to putting photos on social media. Because that is shared with the people that we think are most important. Also, it gets so nicely categorized by month, so you can go back and get an overview of the photos per month and others can comment” (Mother, couple 4). “Then you can scroll back and see when she was a newborn and then go 6 swipes further to see her at 6 months” (Father, couple 4).

One couple even created a private Instagram account for the child on which recognizable photos are shared with family members. Other measures parents apply are that they shield their own accounts as much as possible. Parents find it important that it starts with themselves, that their decision should be in line with their own sharing behavior on social media.

“We had already set the privacy of our social media profile a little bit stricter, which means that those photos can be more protected” (Mother, couple 2).

Also, one of the parents finds it important that the photos are “acceptable”, meaning not nude. Two couples have specific rules for certain grandparents. They are not allowed to post pictures of their grandchildren at all, because the parents feel that the grandparents are not going to understand the consequences. In their opinion, this could compromise the child’s privacy. Therefore, they feel it is better that the grandparents just do not share pictures. Then nothing can be posted that would be, in parents’ opinion, wrong.

“We then always asked not to post a photo. My mother in law would not understand the difference and would put anything online. We have the feeling that, maybe that’s wrong, but she really does share everything” (Mother, couple 6).

Other parents had to make their viewpoint clear, after a family member already put a picture online, which made the parents react as they were not happy about it. This created privacy turbulence between the family members, leading to clarify and renegotiate the privacy boundary, so the family member would not further post pictures of their child online.

“My sister-in-law, she was only two or three years in the family. She probably did not get the message well. We had a conversation with her, but when it was already too late (Mother, couple 6). “Yes, I had a conversation with her. I am usually the bad cop, she’s [referring to the mother of the child] the good cop. I explained the situation, and she understood and accepted it” (Father, couple 6).

### 3.4. Reactions of online contacts

Some parents were surprised by the positive reactions they received to their decision to share their children’s photos unrecognizable on social media. Because this decision deviated from the norm they observed online, they had rather expected negative reactions. However, sometimes they needed to argue their decision.

“I receive often – at least in the beginning – the question “Why is K. not visible?.” Then I answered “Yes, we have decided to keep him as much as possible from social media. If we post something, then unrecognizable.” I then received immediately the reaction “Well I am very proud of my daughter, so I think it is strange that you do this.” Then I reacted “Yes, but it is not because I do not do that, that I am not proud of my child” (Mother, couple 1).

“My parents, for instance, are from another generation. When they see young parents do this, they say “Oh, this is so strange, why are they doing this?” [in a child’s photo, putting something on the face of the child]. They are immediately pigeonholing these people. However, I think, there is so much behind this that people do not see. People think do not be ridiculous by putting an emoji”, but I think “there is so much more behind this decision”, but people do not know that” (Father, couple 5).

Other parents mentioned also they received many questions and reactions as to why they had taken this decision. Comments and questions that came up from family members or friends are for instance: “Why can’t we see his face?” “Is something wrong with your child?” but even “Do you have ugly children?” “Isn’t that maybe a little excessive?” or “Aren’t you proud of your child?”. This makes parents feel like they must constantly justify themselves. They then try to explain why they made this decision. They emphasize in these conversations the risks of sharenting regarding privacy, the child’s development, and specific risks that could be linked with the disclosure of their child’s image online.

“They ask me personally “why did you do that?”. I told them “look, dangers, pedophilia, you name it, on social media” and they said “ah, that’s a good idea that you do that.” And then I also got responses from other people who did not think it was right for people to post a whole life story of their child online. They also said “yes, that will have an impact on the child when it grows up”” (Father, couple 3).

By explaining their decision, some parents got more understanding for it. However, through these critical comments, parents experienced that sharenting is still considered the social norm, and mindful sharenting less known.

“I do not think that all the world should know every moment, act that he can and does well - that all the world should know that. I am very proud of my child. The WhatsApp is red hot here [laughter]. That’s incredibly common with my family, it’s used a lot and we post a lot, absolutely. With very close friends too, but it’s not because I choose not to share it publicly for all the world, that I’m not proud of my child. And I find that a pity that you have made a decision and then you get such reactions from people, then I think that we indeed live in a society where that is absolutely possible and where more people do make a conscious choice - but then again you notice on that level, that it is something that is still relatively unknown” (Mother, couple 1).

Some parents highlighted that especially grandparents did not understand their decision and did struggle with the fact that they could not share their joys of a new grandchild with others online. By contrast, parents experienced that among young people, this critical vision on sharenting becomes more established. It is no longer taken for granted that grandparents put pictures of children online without the parents’ permission. The respondents experience that more and more young parents are aware of the impact of sharenting, which means that they are more likely to choose to share photos of their children unrecognizable to protect their children from sharenting’s potential drawbacks.

“There are a lot of people that react “oh, I’m going to do that too for my child,” and you see they post something about their children, without them being recognizable” (Father, couple 3).

## 4. Discussion

Until now, research on sharenting has merely looked at parents who engage in sharenting and focused on the motives and perceived consequences of sharing personal information of their children online (Blum-Ross and Livingstone, 2017; Latipah et al., 2020; Cino, 2021; Cino and Wartella, 2021). Other research studied sharenting from adolescents’ perspective by investigating how they perceive their parents’ sharenting and how they react to it (Lipu and Siibak, 2019; Verswijvel et al., 2019; Walrave et al., 2022). The present study adds to the literature by focusing on a specific category of parents who engage in, what we have called, mindful sharenting. These parents want to take advantage of some positive aspects of sharenting while, at the same time, minimizing its risks.

As the transition to parenthood is a difficult adjustment period in some adults’ lives, engaging in sharenting offers prospective and young parents possibilities to build social capital with peers going through the same stage of life (Blum-Ross and Livingstone, 2017). Building social capital is crucial in helping parents cope with the challenges that parenthood brings (Bartholomew et al., 2012). This important driver for some parents to engage in sharenting is also confirmed by respondents in the present study. As far as parents’ motives is concerned to engage in mindful sharenting (RQ1) we found that parents try to balance between the opportunities sharenting can offer them, the social pressure they experience to post news about their child, and their objective to protect their child’s privacy. Parents engaging in (mindful) sharenting form their children’s online identities without their (underage) children’s consent. This can have a negative impact on the development of the personality of their children (Verswijvel et al., 2019). As a result, the parents in this study showed a critical and self-reflective attitude towards sharenting. Moreover, parents were found to engage in mindful sharenting to give their children the opportunity to develop their own online identity.

For the participants, this critical attitude stemmed from the lack of control over sharenting content, concerns regarding the privacy rights of the child and allowing the child the choice to create their own online identity. Parents who engage in mindful sharenting also referred to some negative consequences they observed online, or in their social circles, and ask themselves if parents who post identifiable pictures of their child are enough aware of the risks regarding the privacy of the child, digital security risks such as cyberbullying, or the abuse of pictures on child abuse websites. These potential negative consequences have also been highlighted in previous studies (Steinberg, 2017). These situations and observations were highlighted by parents as a ground for their decision (RQ2).

Mindful sharenting was implemented by adopting several strategies to minimize related risks (RQ3). Parents primarily focused on the photographic and spatial context rather than on the children themselves. For example, the child was photographed from a distance. Other couples post only a body part of the child, such as an ear, a foot, or a hand. Furthermore, the child was also often photographed from behind, when looking away from the camera. There were parents who digitally edited the photos by putting emoticons on the face, blurring the face, or cutting off recognizable parts. Finally, parents tried to regulate the sharenting behavior of relatives by renegotiating their privacy boundaries or asking them not longer to share information of their children on social media to keep control over the child’s online identity.

In this digital age, sharenting is seen as the social norm. Some parents’ decision to share children unrecognizable on social media is seen as a departure from the current norm (Siibak and Traks, 2019). New parents feel externally and internally encouraged to share the content of their children online (Blum-Ross and Livingstone, 2017). Firstly, parents are internally stimulated to share images that demonstrate their involvement with their child and their parenting skills. These pictures serve a functional purpose in their self-realization as parents. Secondly, external factors can also play a role in triggering parents to share pictures of their children on social media, showcasing “good parenting,” their proficiency in parenting (Thimm, 2023). For instance, the pressure experienced by parents can be related to the mediatization of everyday life, where both ordinary and extraordinary family experiences are expressed as forming part of the family’s identity. Social media therefore serve as tools to document life experiences and construct a family chronicle (Thimm and Nehls, 2017). In this context, sharenting can also be viewed as a mode of self-expression driven by parents’ inclination for social comparison. By showing positive family moments or their child’s accomplishments, some parents aim also to demonstrate their parenting abilities. In some circumstances, these sharenting posts can be seen as a reaction when comparing oneself as a parent to other parents’ posts concerning their children and the role they play as a parent (Brosch, 2016).

In this context, the decision to engage in mindful sharenting is not taken lightly. This type of sharenting in which parents consciously choose to employ strategies to limit the disadvantages of sharenting, but allows them to enjoy the benefits it offers them, is a difficult balance they want to strike. Mindful sharenting may thus provide an answer to the privacy-openness paradox (Chalklen et al., 2017). The paradox is between parents’ sharing of information about their children on social media to enjoy the benefits of sharenting, and expectations from their relatives and friends, and the privacy and other potential risks they and their children may experience, now or later. Mindful sharenting thus allows parents to enjoy the benefits, but at the same time try to protect their children’s privacy. However, their social circle is not always following them in their decision. While some parents feel supported, others face criticism from those around them, such as “Aren’t you proud of your child?”, “Is something wrong with your child?”, “Isn’t that maybe a little excessive?”. A possible explanation for this contrast between relatives or acquaintances who are supportive or non-supportive for parents’ decision, could be differences in privacy concern or their own value for protecting the child’s privacy in relation to the benefits of sharing identifiable pictures. Also differences in personal privacy breach experiences could explain why some show understanding for, or explicitly support, the decision of the parents to engage in mindful sharenting, while others do not. Future research could therefore focus on possible differences of online contacts’ reactions towards mindful sharenting and find out how this relates to their own privacy concerns, online self-disclosure and related experiences, and how they view possible risks of sharing identifiable pictures of the child. Another possible reason that could be further investigated is related to how relatives react to the privacy boundary determined by the parents. More concretely, parents deciding to publish unidentifiable pictures or restraining online contacts to post or forward child-related pictures may be perceived by online contacts as if they are not allowed into the privacy boundary or withhold their online network—among which friends and relatives—the right to see and share a child’s picture.

Furthermore, the criticism faced by parents engaging in mindful sharenting, made them feel they had to justify their decision, leading some parents to explain why they made this choice by emphasizing the disadvantages and risks associated with sharenting when the child is identifiable.

Next to this conflict concerning their decision to engage in mindful sharenting, parents also try to avoid privacy turbulence (RQ4), as some parents explicitly ask grandparents not to share pictures of their grandchildren or to share pictures only in online conversations but not on publicly accessible online accounts. In terms of CPM, parents are setting privacy boundary linkage and boundary permeability rules. As far as privacy boundary control rules are concerned, we observed some parents want to stay in control of the dissemination of their child’s personal information. Some parents strictly do not offer grandparents autonomy to decide for themselves, even asking them not to take the initiative to share personal information about their grandchild, as some parents think grandparents do not understand the consequences.

In some cases, parents have found themselves needing to clarify their point of view concerning sharing of pictures of their child when a family member had already put a picture online, causing parents to feel unhappy about the situation. This created privacy turbulence, leading to renegotiations of the privacy boundary, namely that the family member would not share pictures of their child. Some parents try to avoid privacy turbulence by sharing recognizable photos with family members and close friends through private WhatsApp groups, to be able to be more selective about the people who have access to the pictures. A few parents use other applications to share photos with selected recipients (e.g., Google Photos, or a private Instagram account). By sharing the pictures in closed groups and by explicitly telling family members that the pictures may not be further disseminated, parents want to prevent misunderstandings and, in terms of the CPM theory, prevent privacy turbulence.

Notwithstanding the present study’s results, several limitations must be acknowledged. Due to the qualitative nature of the study, a limited number of participants were interviewed. The participants were all near or in their 30s. As a result, it was not possible to determine whether ways of engaging in mindful sharenting differed between generations of parents. Future research could examine potential differences in motives and strategies used to engage in (mindful) sharenting. Another limitation of the present study is that both parents were interviewed together. This could have led to social desirability or parent’s avoiding some topics to prevent discussions between partners. Future research could interview parents separately and confront their opinions on how the decision was made to engage in mindful sharenting, how strategies to protect the child’s privacy were discussed, possible disagreements that emerged during these discussions, and how common decisions were made. Moreover, the present study’s findings are based on a limited number of in-depth interviews. The insights of this qualitative research could be further used in follow-up (cross-sectional or longitudinal) survey research to investigate which strategies are employed more or less frequently, how this relates to parents’ privacy concerns and other characteristics such as parenting styles. This would offer more insight into correlates and predictors of parents’ engagement in mindful sharenting in general, and specific strategies in particular. One of the results of this study is also that some parents who choose mindful sharenting received criticism. Future research could use a longitudinal research design to examine how the way of sharenting evolves, depending on the comments or criticism parents receive. Furthermore, the participants of this study had a Belgian origin. It would therefore be interesting to further investigate whether cultural differences can be observed within or between countries concerning sharenting motives and behaviors, and especially parents’ decision to engage in mindful sharenting. Finally, it is worthy to note that this study took place during the COVID-19 pandemic which made some parents decide to engage in sharenting—although they initially did not—to reach out to their friends and family. In this respect, we believe that studying other circumstances under which parents might turn into sharenting practices for family communication (e.g., when relatives live far away) are relevant to investigate in future research.

## 5. Conclusion

Previous research on sharenting has primarily focused on parents’ motivations and their perceived consequences of sharing personal information about their children on social media, as well as adolescents’ perspectives and reactions on their parents’ sharenting behavior. The present study contributes to the literature by focusing on parents who engage in mindful sharenting to leverage the benefits of sharenting while minimizing the potential risks. More particularly, parents’ motives, the privacy protective strategies they use and how relatives and acquaintances react on their decision were investigated. Parents practicing mindful sharenting demonstrate a critical and self-reflective attitude towards sharenting. They want to protect their child’s privacy and avoid that images they post would lead to abuse or aggression, now or later. They are driven by concerns over lack of control, protection of their child’s privacy, and allowing their child to shape their own online identity. Parents were sometimes stimulated to take this decision due to their own or acquaintances’ previous negative experiences when they engaged in sharenting. Mindful sharenting concretely involves parents engaging in new pictorial practices, focusing on the context of the photographs rather than the children themselves. It also includes photographing the child from a distance, capturing only specific body parts, or using digital editing techniques. These protective strategies, however, lead also to discussions with family members and other online contacts, pushing some parents to justify their decision. Moreover, parents’ decision to engage in mindful sharenting is sometimes thwarted by family members who post identifiable pictures as today sharenting has become the social norm, making the decision to engage in mindful sharenting a departure from this norm. In sum, mindful sharenting attempts to strike a delicate balance between reaping the benefits of sharenting while trying to safeguard their child’s privacy and minimize risks.

## Data availability statement

The raw data supporting the conclusions of this article will be made available by the authors, without undue reservation.

## Ethics statement

Ethical review and approval was not required for the study on human participants in accordance with the local legislation and institutional requirements. The patients/participants provided their written informed consent to participate in this study.

## Author contributions

MW was responsible for the research idea, study design, parts of the literature study, and the drafting of major parts of the manuscript. SR was responsible for parts of the literature study and the manuscript, the data collection and data analyses, and proofreading. LS and LH were responsible for parts of the literature study, contributions to the theoretical, methodological and empirical parts of the manuscript, and proofreading. All authors contributed to the article and approved the submitted version.

## Conflict of interest

The authors declare that the research was conducted in the absence of any commercial or financial relationships that could be construed as a potential conflict of interest.

## Publisher’s note

All claims expressed in this article are solely those of the authors and do not necessarily represent those of their affiliated organizations, or those of the publisher, the editors and the reviewers. Any product that may be evaluated in this article, or claim that may be made by its manufacturer, is not guaranteed or endorsed by the publisher.

## References

[ref1] Ammari T. Kumar P. Lampe C. Schoenebeck S. (2015). “Managing Children’s online identities: how parents decide what to disclose about their children online.” in *Proceedings of the 33rd annual ACM conference on human factors in computing systems*. pp. 1895–1904.

[ref2] Autenrieth U. (2018). “Family photography in a networked age. Anti-sharenting as a reaction to risk assessment and behaviour adaption” in Digital parenting. The challenges for families in the digital age, Nordicom, University of Gothenburg. 219–231.

[ref3] Auxier B. Anderson M. Perrin A. Turner E. (2020). Parents’ attitudes—and experiences—related to digital technology. Pew Research Center. https://www.pewresearch.org/internet/2020/07/28/parents-attitudes-and-experiences-related-to-digital-technology/ (Accessed December 1, 2022).

[ref4] Bartholomew M. K. Schoppe-Sullivan S. J. Glassman M. Kamp Dush C. M. Sullivan J. M. (2012). New parents’ Facebook use at the transition to parenthood. Fam. Relat. 61, 455–469. doi: 10.1111/j.1741-3729.2012.00708.x, PMID: 23671354 PMC3650729

[ref5] Binder J. Howes A. Sutcliffe A. (2009). “The problem of conflicting social spheres: effects of network structure on experienced tension in social network sites.” in *Proceedings of the SIGCHI Conference on Human Factors in Computing Systems*. pp. 965–974.

[ref6] Blum-Ross A. Livingstone S. (2017). “Sharenting,” parent blogging, and the boundaries of the digital self. Pop. Commun. 15, 110–125. doi: 10.1080/15405702.2016.1223300

[ref7] Braun V. Clarke V. (2012). “Thematic analysis” in APA handbook of research methods in psychology, Vol. 2: Research designs: Quantitative, qualitative, neuropsychological, and biological. eds. Cooper C. Camic P. M. Long D. L. Panter A. T. Rindskopf D. Sher K. J.. Washington DC: American Psychological Association, 57–71. doi: 10.1037/13620-004

[ref8] Brosch A. (2016). When the child is born into the internet: Sharenting as a growing trend among parents on facebook. New Educ. Rev. 43, 225–235. doi: 10.15804/tner.2016.43.1.19

[ref9] Brosch A. (2018). Sharenting: why do parents violate their Children’s privacy? New Educ. Rev. 54, 75–85. doi: 10.15804/tner.2018.54.4.06

[ref10] Cambridge Dictionary (2023). Anti. Available at: https://dictionary.cambridge.org/ (Accessed January 9, 2023).

[ref11] Chalklen C. Anderson H. Anderson H. M. (2017). Mothering on Facebook: exploring the privacy/openness paradox. Soc. Media Soc. 3:205630511770718. doi: 10.1177/2056305117707187

[ref12] Child J. Petronio S. (2015). Privacy management matters in digital family communication, *in Family Communication in the Age of Digital and Social Media*, eds. C. J. Bruess, Peter Lang, 32–54.

[ref13] Cino D. (2021). The “5 Ws and 1 H” of Sharenting: findings from a systematized review. Italian Soc. Rev. 11:853. doi: 10.13136/isr.v11i3.495

[ref14] Cino D. Dalledonne Vandini C. (2020). “My kid, my rule”: governing children’s digital footprints as a source of dialectical tensions between mothers and daughters-in-law. Stud. Commun. Sci. 20, 181–202. doi: 10.24434/j.scoms.2020.02.003

[ref15] Cino D. Wartella E. (2021). Privacy-protective behaviors in the mediatized domestic milieu: parents and the intra- and extra-systemic governance of children’s digital traces. Ricerche Di Pedagogia e Didattica. J. Theories Res. Educ., 16, 133–153. doi: 10.6092/ISSN.1970-2221/13276

[ref16] Collett J. L. (2005). What kind of mother am I? Impression management and the social construction of motherhood. Symb. Interact. 28, 327–347. doi: 10.1525/si.2005.28.3.327

[ref17] Damkjaer M. S. (2018). “Sharenting = good parenting? Four parental approaches to Sharenting on Facebook” in Digital parenting. The challenges for families in the digital age. Nordicom, University of Gothenburg. 209–218.

[ref18] Davidson-Wall N. (2018). “Mum, seriously!”: Sharenting the new social trend with no opt-out. Debating communities and social networks 2018 OUA conference. Available at: http://networkconference.netstudies.org/2018OUA/wp-content/uploads/2018/04/Sharenting-the-new-social-trend-with-no-opt-out.pdf (Accessed December 1, 2022).

[ref19] Diebel T. (2022). So ein Bild von dir würdest du nie posten? Dein Kind auch nicht. Available at: https://deinkindauchnicht.org/ (Accessed July 21, 2022).

[ref20] Garmendia M. Martínez G. Garitaonandia C. (2021). Sharenting, parental mediation and privacy among Spanish children. Eur. J. Commun. 37, 145–160. doi: 10.1177/02673231211012146

[ref21] Geurtzen N. Scholte R. H. J. Engels R. C. M. E. Tak Y. R. van Zundert R. M. P. (2015). Association between mindful parenting and adolescents’ internalizing problems: non-judgmental acceptance of parenting as Core element. J. Child Fam. Stud. 24, 1117–1128. doi: 10.1007/s10826-014-9920-9

[ref22] Heirman W. Walrave M. Vermeulen J. Ponnet K. Vandebosch H. Van Ouytsel J.. (2016). An open book on Facebook? Examining the interdependence of adolescents\textquotesingle privacy regulation strategies. Behav. Inform. Technol. 35, 706–719. doi: 10.1080/0144929X.2016.1181210

[ref23] Hennink M. Kaiser B. N. (2022). Sample sizes for saturation in qualitative research: A systematic review of empirical tests. Soc. Sci. Med. 292:114523. doi: 10.1016/j.socscimed.2021.114523, PMID: 34785096

[ref24] Holiday S. Norman M. S. Densley R. L. (2022). Sharenting and the extended self: self-representation in parents’ Instagram presentations of their children. Pop. Commun. 20, 1–15. doi: 10.1080/15405702.2020.1744610

[ref25] Kumar P. Schoenebeck S. (2015). “The modern day baby book: enacting good mothering and stewarding privacy on Facebook.. in Proceedings of the 18th ACM Conference on Computer Supported Cooperative Work & Social Computing. pp. 1302–1312.

[ref26] Lampinen A. Tamminen S. Oulasvirta A. (2009). “All my people right Here, right now: management of group co-presence on a social networking site.” in Proceedings of the ACM 2009 international conference on supporting GROUP work - GROUP ’09. p. 281.

[ref27] Latipah E. Kistoro H. C. A. Hasanah F. F. Putranta H. (2020). Elaborating motive and psychological impact of sharenting in millennial parents. Univ. J. Educ. Res. 8, 4807–4817. doi: 10.13189/ujer.2020.081052

[ref28] Leary M. R. (2001). “Impression management, psychology of” in International encyclopedia of the Social & Behavioral Sciences. Amsterdam, the Netherlands: Elsevier, 7245–7248.

[ref29] Leaver T. (2015). Born digital?: presence, privacy, and intimate surveillance. 149–160.

[ref30] Leaver T. (2020). “Balancing privacy: Sharenting, intimate surveillance, and the right to be forgotten” in The Routledge companion to digital media and children. eds. Green L. Holloway D. Stevenson K. Leaver T. Haddon L.. 1st ed. New York:Routledge, 235–244.

[ref31] Lipu M. Siibak A. (2019). ‘Take it down!’: Estonian parents’ and pre-teens’ opinions and experiences with sharenting. Media Int Aust 170, 57–67. doi: 10.1177/1329878X19828366

[ref32] Marwick A. E. Boyd D. (2011). I tweet honestly, I tweet passionately: twitter users, context collapse, and the imagined audience. New Media Soc. 13, 114–133. doi: 10.1177/1461444810365313

[ref33] Otero P. (2017). Sharenting… should children’s lives be disclosed on social media? Arch. Argent. Pediatr. 115, 412–413. doi: 10.5546/aap.2017.eng.412, PMID: 28895685

[ref34] Ouvrein G. Verswijvel K. (2019). Sharenting: parental adoration or public humiliation? A focus group study on adolescents’ experiences with sharenting against the background of their own impression management. Child Youth Serv. Rev. 99, 319–327. doi: 10.1016/j.childyouth.2019.02.011

[ref35] Petronio S. (2002). Boundaries of privacy:Dialectics of disclosure, Albany, New York: State University of New York Press.

[ref36] Petronio S. Child J. T. (2020). Conceptualization and operationalization: utility of communication privacy management theory. Curr. Opin. Psychol. 31, 76–82. doi: 10.1016/j.copsyc.2019.08.009, PMID: 31526974

[ref37] Picone I. (2015). “Impression Management in Social Media” in The international encyclopedia of digital communication and society. eds. Ang P. H. Mansell R.. 1st ed. Hoboken, New Jersey: Wiley, 1–7.

[ref38] Prensky M. (2001). Digital natives, digital immigrants part 1. Horizon 9, 1–6. doi: 10.1108/10748120110424816

[ref39] Putnam R. D. (2000). “Bowling alone: the collapse and revival of American community.” in *Proceedings of the 2000 ACM Conference on Computer Supported Cooperative Work - CSCW ’00*. p. 357.

[ref40] Putri N. R. Harkan A. A. Khairunnisa A. A. Nurintan F. Ahdiyat Moh A. (2021). Construction of “sharenting” reality for mothers who shares Children’s photos and videos on Instagram: Asia-Pacific research in social sciences and humanities Universitas Indonesia conference (APRISH 2019), Jakarta, Indonesia.

[ref41] Ranzini G. Newlands G. Lutz C. (2020). Sharenting, peer influence, and privacy concerns: A study on the Instagram-sharing behaviors of parents in the United Kingdom. Soc. Media Soc. 6:205630512097837. doi: 10.1177/2056305120978376

[ref42] Robiatul Adawiah L. Rachmawati Y. (2021). Parenting program to protect Children’s privacy: the phenomenon of Sharenting children on social media. JPUD 15, 162–180. doi: 10.21009/JPUD.151.09

[ref43] Shorey S. Ng E. D. (2021). The efficacy of mindful parenting interventions: A systematic review and meta-analysis. Int. J. Nurs. Stud. 121:103996. doi: 10.1016/j.ijnurstu.2021.103996, PMID: 34175531

[ref44] Siibak A. Traks K. (2019). The dark sides of sharenting. Catalan J. Commun. Cult. Stud. 11, 115–121. doi: 10.1386/cjcs.11.1.115_1

[ref45] Smahel D. MacHackova H. Mascheroni G. Dedkova L. Staksrud E. Olafsson K.. (2020). EU kids online 2020: survey results from 19 countries. London School of Economics and Political Science. Available at: http://eprints.lse.ac.uk/103294/ (Accessed December 1, 2022).

[ref46] Steinberg S. B. (2017). Sharenting: Children’s privacy in the age of social media. Emory Law J. 66, 839–884.

[ref47] Steuber K. R. McLaren R. M. (2015). Privacy recalibration in personal relationships: rule usage before and after an incident of privacy turbulence. Commun. Q. 63, 345–364. doi: 10.1080/01463373.2015.1039717

[ref48] Thimm C. (2023). “Mediatized families: digital parenting on social media” in Families and new media. eds. Dethloff N. Kaesling K. Specht-Riemenschneider L.. Wiesbaden, Germany: Springer, 33–57.

[ref49] Thimm C. Nehls P. (2017). Sharing grief and mourning on Instagram: digital patterns of family memories. Communications 42, 327–349. doi: 10.1515/commun-2017-0035

[ref50] Tifentale A. Manovich L. (2018). “Competitive photography and the presentation of the self” in Exploring the selfie: Historical, analytical, and theoretical approaches to digital self-photography. Cham, Switzerland: Palgrave Macmillan, 167–187.

[ref51] Verswijvel K. Walrave M. Hardies K. Heirman W. (2019). Sharenting, is it a good or a bad thing? Understanding how adolescents think and feel about sharenting on social network sites. Child Youth Serv. Rev. 104, 104401–104410. doi: 10.1016/J.CHILDYOUTH.2019.104401

[ref52] Wagner A. Gasche L. A. (2018). Sharenting: making decisions about other’s privacy on social networking sites. Retrieved from: https://www.leuphana.de/institute/iis/mkwi2018.html (Accessed December 1, 2022).

[ref53] Walrave M. Vanwesenbeeck I. Heirman W. (2012). Connecting and protecting? Comparing predictors of selfdisclosure and privacy settings use between adolescents and adults. Cyberpsychology, 6. doi: 10.5817/CP2012-1-3

[ref54] Walrave M. Verswijvel K. Ouvrein G. Staes L. Hallam L. Hardies K. (2022). The limits of Sharenting: exploring parents’ and adolescents’ Sharenting boundaries through the Lens of communication privacy management theory. Front. Educ. 7:803393. doi: 10.3389/feduc.2022.803393

[ref55] Williams-Ceci S. Grose G. E. Pinch A. C. Kizilcec R. F. Lewis N. A. (2021). Combating sharenting: interventions to alter parents’ attitudes toward posting about their children online. Comput. Hum. Behav. 125:106939. doi: 10.1016/j.chb.2021.106939

